# Mechanisms and therapeutic potential of collateral sensitivity to antibiotics

**DOI:** 10.1371/journal.ppat.1009172

**Published:** 2021-01-14

**Authors:** Roderich Roemhild, Dan I. Andersson

**Affiliations:** 1 Institute of Science and Technology, Klosterneuburg, Austria; 2 Uppsala University, Department of Medical Biochemistry and Microbiology, Uppsala, Sweden; Nanyang Technological University, SINGAPORE

## What is collateral sensitivity?

Collateral sensitivity (CS) is an evolutionary trade-off between antibiotic resistance mechanisms in bacteria and describes a situation where antibiotic resistance to 1 antibiotic confers increased susceptibility to another antibiotic. CS typically means that inhibition of growth can be achieved with lower concentrations of antibiotic (the strain has a reduced minimal inhibitory concentration (MIC)). For bactericidal antibiotics, CS can also mean the faster and stronger killing of the resistant bacterium, compared with one that does not carry the resistance mechanism [1]. A prominent example of CS is the >16-fold lower MIC of tigecycline-resistant *Escherichia coli* for nitrofurantoin where resistance to tigecycline and sensitivity to nitrofurantoin are caused by a mutation of the *lon* gene [2]. CS has been described for various chromosomal mutations [3], resistance plasmids [4], and resistance genes [5].

It is important to differentiate the genetically based CS effects from related physiological phenomena that cause a similar increase of antibiotic efficacy. Antibiotic synergy refers to the nonlinear increase of inhibition when antibiotics are applied in mixture [6], and negative hysteresis refers to a transient increase of antibiotic efficacy to 1 drug after a short preceding exposure to another drug [7]. In contrast to CS, synergy and hysteresis both involve physiological responses to drug exposure without any associated genetic changes. In this article, we focus entirely on CS, its causative mechanisms, and the therapeutic potential of the phenomenon.

## Why care about CS?

Resistance to antibiotic treatment is increasing due to the continued selection for less-susceptible genetic variants in clinical and nonclinical environments. Resistant pathogens may cause a bacterial infection that is hard to treat, and new resistance mechanisms may emerge during infection as an evolutionary response to antibiotic therapy. The mechanisms by which these variants resist inhibition from antibiotics are quite well understood and include (1) the reduced penetration of the cell envelope; (2) the chemical destruction of antibiotic by specialized proteins; (3) metabolic bypass of antibiotic targets; and (4) the modification of target structures that reduce binding [8]. In contrast, genetic mechanisms that increase the inhibitory effect of antibiotic treatments, as observed for CS, are less understood. When understood, the concept of CS could be applied as a last-resort treatment for pathogens with a matching resistance mechanism. Alternatively, CS could be included in treatment designs as a mechanism to reduce/prevent resistance emergence.

## How does CS work?

**Fig 1:**
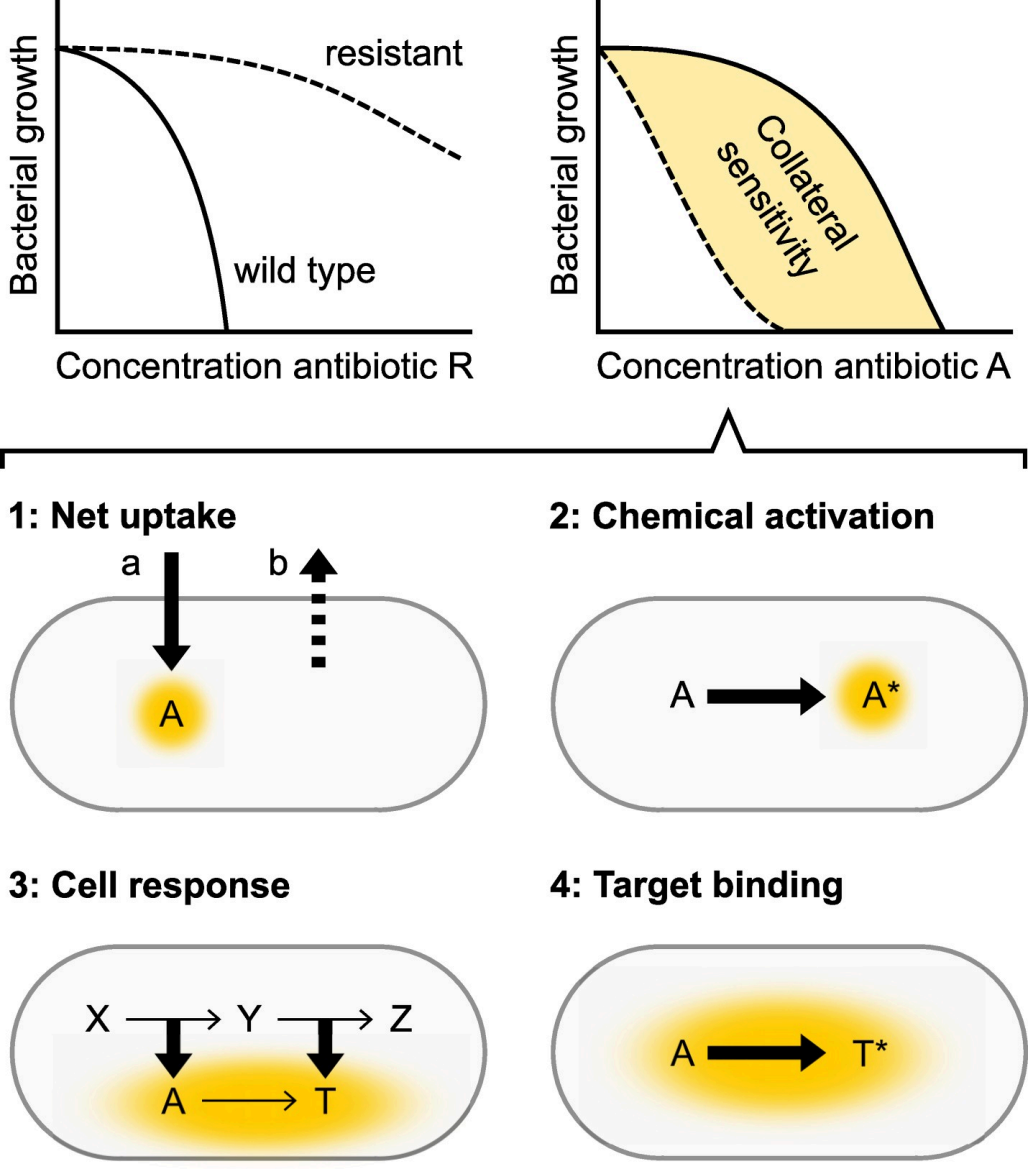
Mechanisms of collateral sensitivity. Schematic illustration of resistance (top left) and collateral sensitivity (top right) of a strain with resistance mechanism against antibiotic *R*. The resistant strain is indicated with a dashed line, and the susceptible wild-type strain with a solid line. Yellow shading indicates increased inhibitory activity of antibiotic *A*. The inhibitory effect of *A* that is generated upon binding of target structure *T* may be increased by several theoretical mechanisms (numbered 1–4). Bold arrows indicate increased functions. Broken arrows indicate reduced functions.

A mechanistic understanding of CS may be inspired by our present knowledge of resistance. Applying the rule of inversion to described resistance mechanisms, we arrive at several potential sensitivity mechanisms (Fig 1). The CS of a resistance mechanism may result from an increased concentration of active antibiotic at the target structure, as attained by either an (1a) increased penetration of the cell envelope; (1b) reduced efflux of the antibiotic; or (2) the formation of chemically more active antibiotic through the activity of specialized proteins. Alternatively, it may result from (3) modified cellular functions and regulatory pathways that increase the toxic effects of the antibiotic, or (4) the increased binding of antibiotic to modified target structures. In addition to these effects, a false-positive CS signal may result from a generally weak growth ability of resistant pathogens even in the absence of treatment (the fitness cost of resistance). Despite abundant theoretical explanations, experimental data are still rare.

The first sensitivity mechanism to be understood is the generally 2- to 4-fold lower MIC of aminoglycoside-resistant *E*. *coli* to several classes of antibiotics (beta-lactams, fluoroquinolones, chloramphenicol, doxycycline, tetracycline) that is caused by mutations in the ion transport protein *trkH* [3]. The uptake of aminoglycoside antibiotics into the cell requires a high proton motive force (PMF), and the mutation in *trkH* reduces the PMF, explaining resistance to aminoglycoside. The activity of the major efflux pump system AcrAB-TolC is also driven by PMF, so that as a lateral effect, the *trkH* mutation reduces the expulsion of several classes of antibiotics, explaining the increased sensitivity through mechanism 1b [3].

However, sensitivity mechanisms can be more specific to the antibiotic. The antibiotic nitrofurantoin is a prodrug that for activity requires chemical activation inside the cell by the nitroreductase enzymes NfsA and NfsB. A recent study could link the CS to nitrofurantoin of mecillinam-resistant *E*. *coli* with mutations in *spoT* to the increased expression of nitroreductase enzyme NfsB [2]. The same mechanism also conferred nitrofurantoin sensitivity in a tigecycline-resistant *lon* mutant of *E*. *coli* and a protamine-resistant *hemL* mutant of *Salmonella enterica* [2]. Intriguingly, several additional sensitivity mechanisms contributed to the total collateral effect in the *lon* and *hemL* mutants. The *hemL* mutant showed increased uptake of nitrofurantoin, according to sensitivity mechanism 1a. The *lon* mutant provided an interesting case of mechanism 3 and deserves a more detailed account.

Activated nitrofurantoin causes damage to DNA, causing cells to activate a native DNA-repair system designated the *lexA*-dependent SOS response [9]. As part of the SOS response, cells produce the SulA protein that stops growth during the repair process by inhibiting the ring polymerization of the cell division protein FtsZ [10,11]. SulA is degraded by the Lon protease [12], and cell growth resumes a short while after the DNA damage is repaired. However, in a *lon* mutant, accumulated SulA is not degraded and, consequently, cells stay locked in the nondividing SOS state, explaining its increased sensitivity to nitrofurantoin. When cells are experimentally equipped with noninducible alleles of *lexA*, they recover approximately two-thirds of their total >16-fold reduction in MIC [2]. This example demonstrates the complexity of CS mechanisms.

Finally, recent work provides an example for mechanism 4 [5]. The beta-lactamase gene CTX-M-15 is present in several clinically relevant resistance plasmids. Beta-lactamases are enzymes that can hydrolyse the antibiotic and chemically inactivate it. The mutation N135D in this gene increases the MIC to the beta-lactam mecillinam 50-fold, but decreases the MIC to cefotaxime, a slightly different beta-lactam, 20-fold [5]. The linked changes in sensitivity are likely explained by noncompatible adjustments of the antibiotic binding site in the protein [5]. Together, these studies provide a glimpse at the fascinating genetic mechanisms behind CS. They highlight how research of CS can increase our general understanding of bacterial physiology.

## How to use CS?

One key question regarding CS is how one could potentially exploit it in clinical settings to increase efficacy and/or reduce resistance evolution. Since CS has several potentially useful effects, e.g., (1) inhibition of growth with lower concentrations of antibiotic and (2) faster and stronger killing of a resistant bacterium (compared to the susceptible), it could be applied to both increasing efficacy and/or reducing the rate of resistance evolution. One potential application are antibiotics where toxicity is an issue (e.g., aminoglycosides, vancomycin, colistin), and the drug concentration should be kept as low as possible without compromising efficacy. Thus, in cases when the infection is caused by a bacterium that carries a resistance to another drug that increases susceptibility to any of these toxic antibiotics (which is expected be quite rare), a lower concentration could be used for treatment. The second, more conceivable clinical application that has received the most interest, is to use 2 drugs which show CS (preferably reciprocal CS; i.e., resistance to antibiotic 1 increases susceptibility to antibiotic 2 and vice versa) in combination or sequentially in a temporal cycling scheme to reduce the rate of resistance evolution. Several studies have shown that both combination or sequential use can reduce the rate of resistance evolution under specific laboratory conditions [13–15]. However, in vitro studies also highlighted variability of the evolutionary trade-off between experimental replicates (e.g., [16–18]). While the average trade-off was CS, for certain antibiotic pairs, the phenotype of independent replicates varied from strong CS to cross-resistance, as explained by the fixation of alternative resistance mechanisms or alleles [16–18]. The resulting collateral variation is expected from the stochastic supply of mutations, especially under small population sizes and wide mutation spaces [16], and may considerably complicate the utility of CS for therapy.

## Is CS clinically applicable?

For CS to become more than an interesting example of an evolutionary trade-off, it needs clinical testing. There are several examples of multidrug therapies that are in clinical use, e.g., for HIV, malaria, and tuberculosis infections, demonstrating their utility for certain types of infections. However, the effectiveness of these combinations is not connected to CS per se (even though it might inadvertently occur) but rather with a general suppression of the likelihood of resistance evolution and/or increasing efficacy by combining antibiotics with different pharmacodynamic/pharmacokinetic properties.

To our knowledge, no clinical study has prospectively tested the impact of CS on resistance evolution, but a few papers have measured resistance development (mostly in *P*. *aeruginosa*) under laboratory conditions and inferred expected clinical effects. One important study found agreement of laboratory-evolved CS with the population dynamics of *P*. *aeruginosa* resistance mutations in a hospitalized patient [19]. The monitored infection contained several resistant subpopulations, whose relative frequency changed dramatically during treatment and, importantly, as expected from lab-evolved CS in the PAO1 strain and several clinical strains [19]. This case example indicates an overlap of resistance dynamics during clinical treatment and laboratory “forecasting.” Comparative laboratory adaptation of *P*. *aeruginosa* with 38 different drug mixtures showed that slow adaptation was significantly associated with CS [13], and another study [14] showed that a CS treatment of resistant clones selects for new resistance mechanisms that can disrupt the first resistance mechanism, causing resensitization in alternating treatments. In this case, the resensitization depended on treatment order and was characteristic for aminoglycoside to beta-lactam switches, but not the reverse switches [14]. A study of tobramycin-ceftazidime showed that ceftazidime selects pyomelanogenic tobramycin-hypersusceptible mutants and that this trade-off allows tobramycin/ceftazidime alternation to drive extinction of resistant populations [15]. The evolution of CS is also dependent on the environment as shown by a comparison of *Acinetobacter baumannii* evolving resistance to ciprofloxacin in culture versus biofilm [20] where CS to cephalosporins was only observed under evolution in biofilms.

In contrast to the above studies, there is also evidence for the absence of useful evolved CS patterns in clinical isolates. One study showed for isolates adapted to antibiotics in the lung of cystic fibrosis patients [21] that resistant clinical isolates do not show consistent sensitivities to other antibiotics, as is usually seen for vitro evolution experiments. The explanation for this discrepancy is unclear, but it could be related to differences in antibiotic selective pressures and mutational spectra or that adaptation to other selective pressures in vivo abrogates CS and makes it evolutionary transient.

Based on the above finding, a priori one can imagine a number of complications that could reduce the usefulness of CS and that need to be studied. These include (1) the effect size (how much is sensitivity increased), which is generally quite small, <5-fold [1,3], even though the effects can also be more pronounced [22]; (2) the evolutionary conservation of CS (or lack thereof) [23,24] within a species; (3) the lack of data showing its occurrence for horizontally transferred genes (prior studies focused on mutational resistance); (4) the prevalence and effectiveness of various types of escape mutants that could abrogate CS [14,21]; and (5) the clinical relevance of the combinations of antibiotics and resistance mechanisms that have been studied during laboratory experiments (e.g., the toxicity of certain drugs that are tested in laboratory settings constrains their clinical application) [21,23].

In conclusion, the phenomenon of CS shows a number of useful characteristics under laboratory conditions and has revealed several novel links between different resistance mechanisms and antibiotic action. However, the clinical application of CS remains unclear, and addressing it will require prospective clinical treatment studies to examine its ability to increase efficacy and/or reduce the rate of resistance development. Until such data are available, CS will remain an interesting biological phenomenon in search of a clinical application.
